# Numerical Simulation and Prediction of Flexure Performance of PSC Girders with Long-Term Prestress Loss

**DOI:** 10.3390/ma18204654

**Published:** 2025-10-10

**Authors:** Jun-Hee Won, Woo-Ri Kwon, Jang-Ho Jay Kim

**Affiliations:** School of Civil and Environmental Engineering, Yonsei University, 50, Yonsei-ro, Seodaemun-gu, Seoul 03722, Republic of Korea; wjhbaram@yonsei.ac.kr (J.-H.W.); uskwon@yonsei.ac.kr (W.-R.K.)

**Keywords:** flexural behavior, post-tension, PSC girder, Finite Element Method (FEM) simulation

## Abstract

The purpose of this parametric study was to develop a numerical simulation model calibrated with experimental data to predict the flexural behavior of prestressed concrete (PSC) girders subjected to long-term prestress losses. The model is capable of accurately simulating the flexural behavior of PSC girders using commercial finite-element (FE) software in the ABAQUS/Explicit program. The accuracy of the model was validated by comparing its results with flexural response test data from three post-tensioned girders, with the tendons ultimately having tensile strength capacities of 1860 MPa, 2160 MPa, and 2400 MPa. The comparison demonstrated generally excellent agreement between numerical and experimental results in terms of the load–deflection response and crack propagation behavior, from the onset of first cracking through the maximum load and into the ductile response range. Subsequently, a parametric study was conducted to evaluate the effects of tendon ultimate strength, amount of long-term prestress loss, grouting defects, degradation-induced reductions in concrete strength, and reductions in tendon cross-sectional area on girder flexural behavior. Through this parametric investigation, the study identified key factors with respect to long-term prestress loss that may influence the flexural behavior of aging PSC structures.

## 1. Introduction

Prestressed concrete (PSC) structures are widely utilized in civil infrastructure, such as bridges, due to their excellent strength and durability that can withstand long-term service. However, over decades of use, these structures undergo various forms of performance degradation, including the gradual loss of prestress over time. Such prestress loss can be induced by factors such as creep and drying shrinkage of concrete, stress relaxation of the prestressing steel, inadequate grouting of tendons, corrosion of strands due to environmental exposure, and the accumulation of micro-damage during construction and maintenance. Since prestressing is one of the primary load-resisting mechanisms in PSC structures, cumulative loss can lead to reductions in flexural strength and deflection resistance, thereby significantly affecting the overall structural stability. In particular, even if a structure appears externally sound, substantial internal prestress loss may have occurred. Therefore, understanding the structural behavior and characteristics that account for such degradation and potential damage is of critical importance. The behavior of such structures can be evaluated through experimental studies or predicted using finite element method (FEM) modeling [1,2,3,4,5,6,7].

### 1.1. Literature Review

Many experimental programs have characterized the behavior of prestress loss or deteriorated PSC members. Garber et al. [7] measured long-term losses on full-scale girders and linked them to stiffness degradation; Limongelli et al. [8] showed systematic reductions of stiffness and natural frequencies with progressive damage in a post-tensioned beam. Regarding corrosion and grouting defects, Zhang et al. [9] observed brittle failure in bonded PT beams with tendon corrosion, while Wang et al. [10] demonstrated that insufficient grouting and corrosion significantly reduce flexural resistance. For fire, Liu et al. [11] tested full-size fire-damaged girders and found notable decreases in residual prestress and flexural strength; a Virginia Transportation Research Council (VTRC) study on field-removed corroded girders reported comparable trends [12]. Together, these data confirm the critical influence of corrosion, grouting defects, and fire on PSC behavior and failure modes.

Parallel efforts have focused on measuring and predicting long-term prestress losses. Guo et al. [13] combined sensors to decompose losses in post-tensioned beams; Kim et al. [14,15] used fiber-optic “smart strands” to validate short- and long-term losses in practice. Han et al. [16] proposed an interaction-based formulation for shrinkage–creep–relaxation. Design provisions are given in AASHTO LRFD [17] and Eurocode 2 [18], while the RILEM B4 model [19] and specialized formulations for unbonded systems [20] and LFBG-based loss decomposition [21] address limitations of code simplifications and regional variability.

FEM studies that embed long-term loss and deterioration are now widespread. Lou et al. [22] presented a nonlinear, time-dependent analysis for continuous unbonded PC beams; Yuen et al. [23] developed and validated a DFEM for a precast segmental bridge with external unbonded tendons, quantifying how prestress variations affect strength and ductility. Galano et al. [24] simulated post-tensioned girders with different prestressing levels and extracted the trade-off between capacity and ductility; Gong et al. [25] modeled time-varying corrosion due to grouting defects and proposed performance-degradation indicators. Accurate simulation of load–deflection and cracking demands consistent use of CDP concrete laws [26,27], fracture-mechanics-based softening [28], and XFEM for crack propagation [29].

### 1.2. Objective

Although numerous studies have investigated prestress loss and performance degradation in prestressed concrete (PSC) girders, studies on tracking and monitoring long-term prestress losses in girders exposed to actual environmental conditions over extended periods, followed by verification of numerical models to assess the resulting effects on girder behavior, have been lacking. In particular, existing studies on long-term prestress loss have primarily focused on measuring the amount of prestress loss and comparing it with existing predictive equations. Moreover, most previous studies have been limited to relatively short durations, typically less than five years, and to a single tendon strength level of 1860 MPa. Therefore, there is a need to expand and diversify numerical analysis models and parameters to account for changes in tendon strength due to the development of high-strength strands, unforeseen defects arising from structural aging, and the cumulative effects of prestress loss over long periods [30,31].

In this study, a finite element model (FEM) was developed for post-tensioned PSC girders that had been exposed to actual environmental conditions for ten years, during which prestress loss occurred. Using the finite element method, experimental results for flexural strength, load–deflection behavior, and tendon strain obtained from these girders were compared with the results of the developed model implemented in the ABAQUS/Explicit 2024. The prestress loss accumulated over the ten-year period was incorporated into the analysis using experimental data reported by Park et al. [32] and a newly modified predictive equation for prestress loss proposed in this study [33,34].

The significance of the proposed model lies in its applicability beyond the ten-year exposure case, enabling future parametric studies on PSC structures that have undergone decades of aging, incorporating prestress loss and various structural defects. Although PSC systems have been refined over decades of development in design methodologies, understanding the changes in structural behavior due to aging remains limited. The main factors contributing to such behavioral changes include prestress loss, grouting defects, and changes in strand strength associated with the development of high-strength prestressing steel. However, experimental testing for each of these factors requires substantial time and financial resources.

The primary objective of this study is to establish a foundation for numerically analyzing the influence of prestress loss and structural defects on the behavior of PSC structures using a finite element model validated through experimental testing. The proposed model is expected to be applicable to various damage scenarios for conducting comprehensive parametric studies, thereby contributing to long-term safety assessment of PSC structures and to the development of performance-based maintenance strategies.

## 2. Experimental Study

### 2.1. Specifications of PSC Specimens

**Figure 1:**
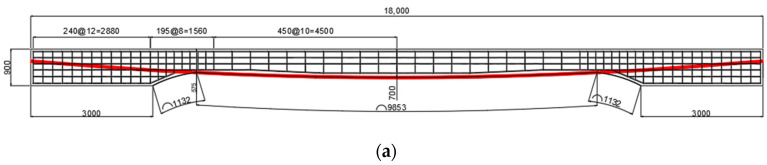

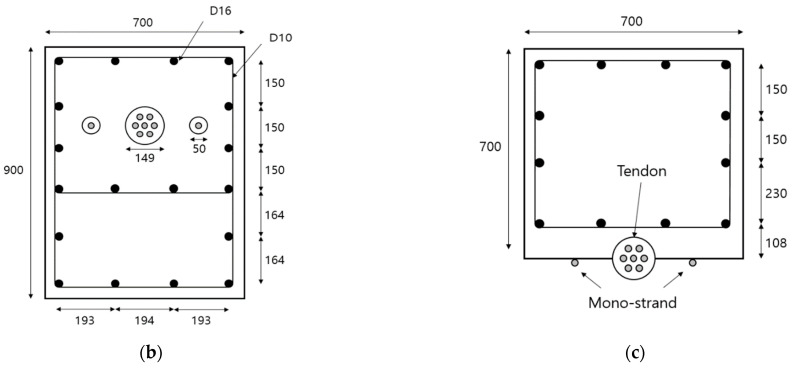
Detail of cross-sectional PSC-girder specimens: (**a**) Front view; (**b**) Cross-section at the anchorage zone; (**c**) Cross-section at midspan.

In this study, a prestressed concrete (PSC) girder with a span of 18 m was fabricated to efficiently measure and analyze long-term prestress loss. The midspan region was designed as a variable-depth section with uniform curvature, providing cross-sectional dimensions of b × h = 700 mm × 900 mm near the supports and 700 mm × 700 mm at midspan. The uniformly curved variable-depth design was adopted to ensure the instrumentation and exposure conditions required for long-term outdoor monitoring of prestress loss. The cross-section of the specimen is shown in Figure 1.

**Figure 2:**
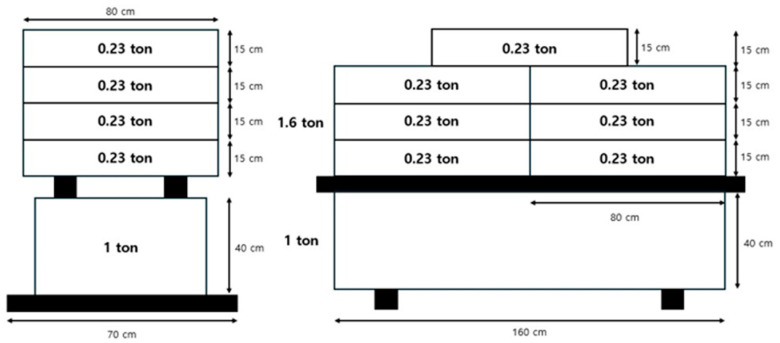
Load-bearing block.

Concrete with a nominal compressive strength of 40 MPa was used, exceeding the minimum requirement for post-tensioned construction ($f_{c}^{\prime }\;\geq$ 30 MPa). Prestressing strands complied with KS D 7002 [35] (types SWPC 7B/7C/7D with ultimate tensile strengths of 1860/2160/2400 MPa), and all had a diameter of 15.2 mm and a modulus of 200 GPa. In the central region, bonded (grouted) tendons were installed with the same curvature, using 7/6/5 strands for the B/C/D types, respectively. For long-term loss monitoring, one exposed monitoring strand was placed symmetrically on each side (two in total) in continuous contact with the girder surface, enabling strain-gauge installation and continuous measurement of prestress loss. Detailed tendon specifications and initial prestressing levels are summarized in Table 1. To apply a sustained load to the specimens, concrete load blocks providing approximately 25.5 kN were placed at the midspan of each girder, as shown in Figure 2 [32].

### 2.2. PS Loss Monitoring Test

**Figure 3:**
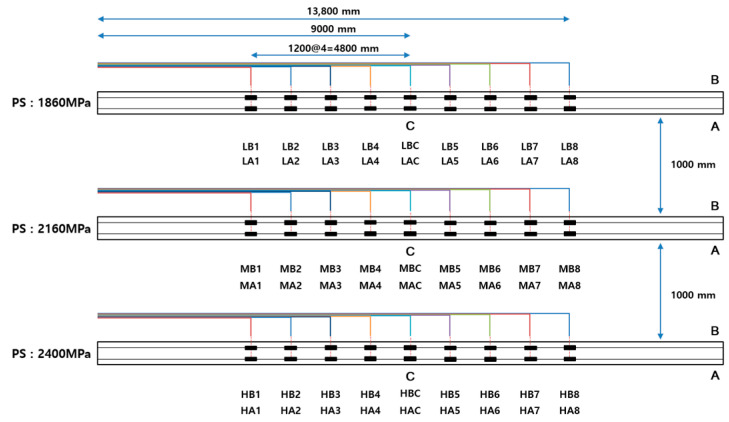
Layout of Strain Gauges for PS Loss Measurement.

To quantify long-term prestress loss, strain gauges (FLA-1-11, Tokyo Measuring Instruments Laboratory/Tokyo Sokki kenkyujo Co., Ltd., Tokyo, Japan) were attached at 1.4 m intervals to two exposed monitoring tendons arranged symmetrically on the left and right. At locations A and B, identical sensors were installed redundantly on both mono tendons so that measurements could be substituted immediately in the event of wire breakage or malfunction. Prior to installation, surface preparation, epoxy bonding, and waterproofing/anticorrosion treatments were performed; sensors were periodically replaced in accordance with adhesive service life, and the initial strain was aligned to the previous reading at each replacement to maintain continuity of the time series. The gauge layout is presented in Figure 3.

Data acquisition was performed with a 50-channel system consisting of a TDS-303 (Tokyo Measuring Instruments Laboratory Co., Ltd., Tokyo, Japan) and a switch box. Measurements were automatically recorded at 00:00, 06:00, 12:00, and 18:00 each day and transmitted remotely. The monitoring period was from January 2015 to June 2024. To assess environmental effects inherent to outdoor monitoring, temperature and humidity data from a nearby meteorological station were managed in parallel [32].

Long-term prestress loss was computed using the highway bridge design code’s long-term loss equation and compared with values measured at midspan over 9 years and 6 months. For the B-type tendon with a nominal strength of 1860 MPa, calculated and measured values agreed reasonably well at representative annual checkpoints. By contrast, for the higher-strength C-type (2160 MPa) and D-type (2400 MPa) tendons, the discrepancy increased from the mid to late stages, indicating that the code tends to systematically overestimate loss. Accordingly, a simple correction is proposed that multiplies the code-calculated loss by a strength-dependent factor V. The correction factor V is defined as the ratio of the initial prestress of the B-type tendon to that of the higher-strength tendon. Using the corrected equation to compute prestress, linear regression analysis showed very high agreement with the measurements. The measured values and the values computed using the modified equation are presented in Table 1. The newly proposed long-term loss prediction is summarized in Equations (1)–(3).

(1) $$V\times ∆f_{p,c+s+r\;}$$

(2) $$V=\frac{f_{pi,L}}{f_{pi,H}}$$

(3) $$∆f_{p,c+s+r}=\frac{\varepsilon_{s}\left(t,t_{0}\right)E_{p}+0.8∆f_{pr}+\alpha \emptyset \left(t,t_{0}\right)(f_{c\left(g+q\right)}+f_{cpo})}{1+\alpha \frac{A_{p}}{A_{c}}\left(1+\frac{A_{c}}{I_{c}}z_{cp}^{2}\right)[1+0.8\left(t,t_{0}\right)]}$$

In Equation (3), the term $\varepsilon_{s}\left(t,t_{0}\right)\;$refers to the shrinkage strain over time, while $E_{p}\;$denotes the modulus of elasticity of the prestressing steel. The term $∆f_{pr}$ accounts for the stress loss due to relaxation, and $\emptyset \left(t,t_{0}\right)$ is the creep coefficient from the time of prestress transfer to the present. The stresses $f_{c\left(g+q\right)}$ and $f_{cpo}$ represent the concrete stress at the steel location due to sustained and initial loads, respectively. The coefficient $\alpha =\;E_{p}/E_{cm}$ considers the relative stiffness between steel and concrete. The denominator includes terms such as $A_{p}$ and $A_{c}$, representing the cross-sectional areas of the prestressing steel and concrete, respectively; $I_{c}$ is the moment of inertia of the concrete section; and $z_{cp}$ is the distance between the centroids of the concrete and tendon. This comprehensive formulation captures the complex long-term prestress loss due to the combined effect of creep, shrinkage, and relaxation. For more detailed information on this equation, refer to the Korean Highway Bridge Design Standard (Limit State Design Method) [36].

### 2.3. Flexural Test

Four-point bending tests were conducted in accordance with KS F 2408 [37] to evaluate the residual strength of the specimens. The tests were performed under displacement control. The loading points were positioned 800 mm from midspan on both sides, and the supports were placed 550 mm from each end of the girder. Loading was applied using a structural testing machine (Shimadzu Corporation, Kyoto, Japan) with a capacity of 5 MN and a maximum displacement range of 450 mm.

To monitor the flexural response, strain gauges were installed on both the concrete and the prestressing tendons, and the readings were continuously recorded with a data logger. The gauge length was 60 mm for the concrete gauges and 1 mm for the tendon gauges, and all gauges were bonded with Cyanoacrylate (CN) adhesive. Girder deflections were measured using Linear Variable Differential Transformer (LVDT) (Tokyo Measuring Instruments Laboratory Co., Ltd., Tokyo, Japan), with two at midspan and one beneath each loading point for a total of four. Since the tests were displacement-controlled, the LVDT data were primarily used to verify measurement accuracy. Strain gauges and LVDTs were installed redundantly and symmetrically on the front and rear surfaces, designated as CA1–CA8 and A1–A8 at the front and CB1–CB8 and B1–B8 at the rear.

For safety and to identify crack initiation, the loading rate was set to 2 mm/min at the beginning. After the midspan deflection reached 100 mm, the LVDTs were removed, and the rate was increased to 5 mm/min. During the observation of the ultimate flexural behavior of the PSC girders, the test was stopped at the ductile stage due to safety concerns related to possible tendon rupture. 

The flexural test results are presented in Table 2 [32]. All terms in Table 2 are defined as follows: $\;P_{cr}$ denotes the first cracking load (kN), and ${∆}_{cr}\;$ is the corresponding deflection (mm). $P_{max}\;$indicates the maximum load recorded during the test (kN), while ${∆}_{max}\;$ is the deflection at maximum load (mm). $K_{i}\;$represents the initial stiffness, calculated as the slope of the load–deflection curve prior to cracking, and $K_{p}\;$ is the post-cracking stiffness, calculated as the slope of the load–deflection curve after cracking.

## 3. FE Simulation Modeling

### 3.1. Tested Specimen Details

**Figure 4:**
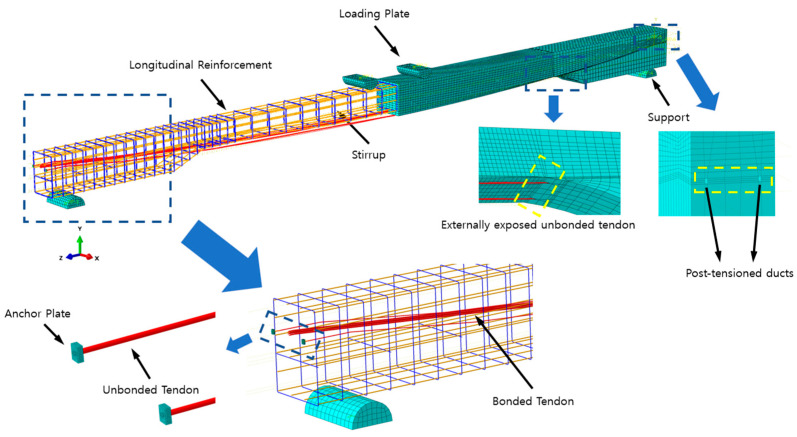
Details of the FE model schematic of a PSC girder.

Detailed configuration of the test specimen used for the four-point bending test of a simply supported PSC girder is shown in Figure 4. The target compressive strength of concrete was designed as 40 MPa. The central portion of the girder, excluding the end support regions, was designed with a tapered curved section to expose the internal prestressing tendons. This configuration allowed the measurement of long-term prestress losses over the past ten years prior to the flexural performance test. Accordingly, the specimen was designed with a uniform curvature, and the concrete thickness gradually decreases with distance from the midspan toward the supports. The cross-section at the supports is 700 mm × 900 mm, whereas at midspan it is 700 mm × 700 mm.

The shear reinforcement consisted of D10 stirrups (10 mm in diameter), and both tensile and compressive longitudinal reinforcements were provided using D16 bars (16 mm in diameter), each with a nominal yield strength of 400 MPa. Three types of prestressing tendons were used in the specimens: SWPC 7B (B-type, 1860 MPa), SWPC 7C (C-type, 2160 MPa), and SWPC 7D (D-type, 2400 MPa). All tendons were assigned a uniform elastic modulus of 200,000 MPa. A total of three girders were fabricated using each of the three high-strength tendon types, and all other structural and material details were kept identical except for the tendon strength. For further details on the girder design and fabrication, refer to Park et al. [32].

### 3.2. Simulation Specimen Modeling Details

The PSC girders post-tensioned with high-strength prestressing tendons were modeled using ABAQUS/CAE 2024. The material properties, specimen dimensions, boundary conditions, and loading conditions in the finite element (FE) model were identical to those used in the experiment. Nonlinear finite element analysis was conducted using ABAQUS/Explicit solver. To replicate the experimental loading protocol, the FE model was subjected to a two-stage loading process: (1) the prestressing force was applied uniformly to the truss elements representing the tendons, and (2) To minimize dynamic effects in the simulation model and promote quasi-static response, a displacement-controlled loading scheme is adopted. The prestressing force was implemented via thermal expansion using the temperature-induced prestress method proposed by Stavroulaki et al. [38].

In terms of element types, the concrete components were modeled using 8-node linear brick elements (C3D8R) with reduced integration and hourglass control. The reduced integration technique was adopted to prevent shear locking and to improve computational efficiency, particularly in large deformation analyses. However, since reduced integration elements may cause spurious zero-energy deformation modes, known as hourglass modes, hourglass control was applied concurrently to suppress such nonphysical behavior and to ensure numerical stability The anchorage plates and the loading/support plates were also modeled using the same element type. Reinforcement bars and stirrups embedded in the concrete were modeled using 2-node truss elements (T3D2), which simulated full bond behavior between concrete and steel. Prestressing tendons were modeled using 1D truss elements (T3D2) for both fully bonded and unbonded/partially bonded cases. In the fully bonded condition, the tendons were considered to be perfectly integrated into the surrounding concrete matrix, while the unbonded or partially bonded cases were represented by T3D2 strands with the appropriate contact/constraint definitions to reflect reduced or absent bond [39,40].

**Figure 5:**
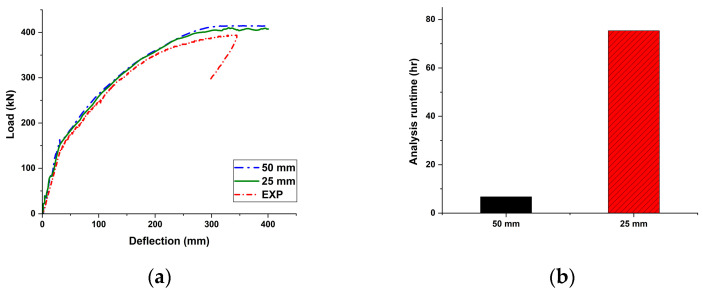
Comparison of load–deflection responses and analysis runtime according to mesh size.: (**a**) Load–deflection curve; (**b**) Runtime required for analysis (black bar: 50 mm mesh; red bar: 25 mm mesh).

To better capture crack patterns and local strain near peak loads, finer mesh sizes were applied in the high-stress midspan region. A mesh size of 50 mm was used in the critical zone, and 100 mm in the remaining concrete regions. This mesh resolution was determined through a mesh sensitivity study, where five different element sizes (300 mm, 200 mm, 100 mm, 50 mm, 25 mm) were simulated. Results indicated that while the 25 mm mesh provided slightly higher resolution, its outcomes were nearly identical to the 50 mm mesh model in terms of the load–deflection response, as shown in Figure 5a. However, the computational time for the 25 mm mesh was approximately 11 times longer than that for the 50 mm mesh, as illustrated in Figure 5b. Therefore, 50 mm mesh was selected for efficiency. Prestressing tendons and reinforcements were meshed with 40 mm elements, while anchorage and loading plates were meshed with 100 mm elements.

#### 3.2.1. Interaction and Constraint

Bonded tendons, stirrups, and longitudinal reinforcement were assumed to be perfectly bonded to the surrounding concrete girder and were implemented using the Embedded Region constraint. The interactions between the unbonded tendons, the empty (ungrouted) ducts, and the soffit of the concrete girder were modeled with a “surface-to-surface” contact. For the normal behavior, “hard” contact with the default constraint enforcement (Lagrange multiplier) was adopted. This hard-contact formulation prevents penetration of the slave surface (the unbonded tendon) into the master surfaces (the duct and the girder soffit) under compression and does not transmit tensile tractions across the interface. With respect to tangential behavior, a Coulomb-type friction model was considered; however, a frictionless condition was adopted to simplify the analysis. This choice is supported by prior studies reporting that the friction between unbonded tendons and ducts is negligible and has little influence on tendon stress development or overall beam behavior [41,42,43,44,45].

#### 3.2.2. Tendon Anchorage Modeling

The anchorage region was modeled using coupling constraints. The bearing face of the anchor plate (defined as an element-based surface) was connected to a reference point (RP) located near the centroid of the face via a distributing kinematic coupling so that reactions are averaged over the entire face. The tendon was represented with T3D2 (truss) elements, and its terminal node (node-based selection) was kinematically coupled to the same RP with displacement degrees of freedom only, since truss elements do not possess rotational degrees of freedom. This arrangement mitigates local stress singularities and avoids over-constraint, while providing a single control/measurement point at the RP for consistent extraction of forces and displacements [46].

#### 3.2.3. Prestressing Force Modeling

The prestressing force was applied using a temperature-induced expansion method, following the approach proposed by Stavroulaki et al. [38]. This indirect modeling technique was developed to overcome the limitations of directly applying initial prestressing loads in general-purpose finite element analysis programs. The method simulates the prestress effect by artificially inducing thermal expansion in the tendons. The linear thermal expansion coefficient of the tendon was defined as $\alpha =1.2\cdot 10^{-5}$ and the corresponding temperature increment (∆T) required to induce the desired prestressing force was calculated by Equation (4) [39].

(4) $$∆T=\frac{f_{ps}}{E\times \alpha }$$

where $f_{ps}$ is the prestressing force; E is Young’s modulus of the tendon; *α* is the coefficient of thermal expansion. The calculated ∆T was applied to the tendon elements as a thermal load, which effectively generated the same mechanical restraint as actual prestressing in the structural analysis. The prestressing force was applied over a separate analysis step with an appropriate loading duration.

As discussed in Section 2.2, the prestressing forces applied for model calibration were adjusted to account for the actual long-term losses that occurred during 10 years of environmental exposure. Accordingly, the prestress for each tendon strength level was rationally determined using the newly proposed long-term loss prediction equation by Park et al. [32] (Equations (1) and (2)). Furthermore, this equation was employed to perform a parametric analysis considering long-term prestress losses beyond 10 years (25, 50, and 100 years), with detailed results presented in Section 6.1. Table 3 provides the tendon specifications, including the correction factor V and the residual prestress values after 10 years of loss.

### 3.3. Material Properties

#### 3.3.1. Concrete CDP

**Figure 6:**
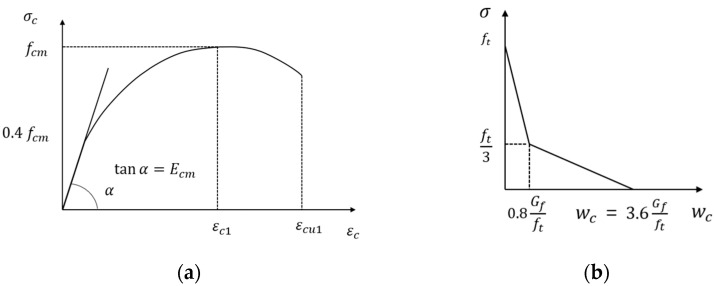
Constitutive model for concrete: (**a**) Compression behavior; (**b**) Tensile behavior.

The behavior of concrete is simulated using the Concrete Damaged Plasticity (CDP) model available in ABAQUS program. This constitutive model is based on a modified version of the Drucker–Prager criterion, originally proposed by Lubliner et al. [26]. and later refined by Lee et al. [27]. It is capable of independently representing tensile cracking and compressive crushing of concrete under monotonic, cyclic, and dynamic loading conditions. To appropriately model concrete behavior, it is essential to account for nonlinear material responses. Under compressive loading, the behavior includes plastic hardening followed by stiffness degradation in the post-peak softening regime. The plastic regime is preceded by an initial linear elastic stress–strain relationship. The uniaxial material model for concrete was formulated in accordance with Eurocode 2 and is presented in Figure 6. The compressive response was determined using the uniaxial stress–strain relationship given in Eurocode 2, Equations (5) and (6), and is shown in Figure 6a [18,47].

(5) $$\frac{\sigma_{c}}{f_{cm}}=\frac{k\left(\varepsilon_{c}/\varepsilon_{c1}\right)-{\left(\varepsilon_{c}/\varepsilon_{c1}\right)}^{2}}{1+\left(k-2\right)\left(\varepsilon_{c}/\varepsilon_{c1}\right)}$$

(6) $$k=1.05\frac{E_{cm}\left|\varepsilon_{c1}\right|}{f_{cm}}$$

The tensile behavior of concrete was defined by applying the fracture energy concept proposed by Hillerborg [33,34]. In this approach, a stress–displacement curve is used instead of a conventional stress–strain curve, as shown in Figure 6b. The ABAQUS Manual indicates that fracture-energy-based modeling can effectively mitigate excessive mesh sensitivity in practical analyses. The fracture energy $G_{f}$ is an intrinsic material property, typically about 40 N/m for normal-strength concrete and up to 120 N/m for high-strength concrete. Hillerborg also noted that, unlike many other material parameters, $G_{f}$ does not require high-precision measurement and exhibits low sensitivity. In the present analysis, $G_{f}$ = 40 N/m was adopted. The tensile strength was evaluated using Eurocode 2 [18], Equation (7).

(7) $$f_{t}=0.3\;{f_{ck}}^{2/3}$$

In addition to defining properties under compression and tension, the CDP model requires several parameters based on the Drucker–Prager hypothesis. The plastic flow potential function and yield surface are formulated using two stress invariants of the effective stress tensor, namely the hydrostatic pressure $\bar{p}$ and the von Mises equivalent effective stress $\bar{q}$, as defined in Equations (8) and (9).

(8) $$\bar{p}=-\frac{1}{3}trace\left(\bar{\sigma }\right)\;\;\;\;\;with\;\;\bar{\sigma }=D_{0}^{el}:(\varepsilon -\varepsilon^{pl})$$

(9) $$\bar{q}=\sqrt{\frac{3}{2}\left(\bar{S}:\;\overset{̿}{S}\right)}\;\;\;\;\;with\;\;\bar{S}=\bar{\sigma }+\bar{p}I$$

where $\bar{S}$ is the deviatoric stress tensor; $\bar{\sigma }$ is the stress tensor; *I* is the identity tensor; $D_{0}^{el}$ is the initial (undamaged) elasticity matrix; ε and $\varepsilon^{pl}$ are the total and plastic strains, respectively. The flow potential function *G* is defined using a hyperbolic Drucker–Prager function, as shown in Equation (10).

(10) $$G=\sqrt{{(\epsilon \sigma_{to}\tan\psi )}^{2}\;+\bar{q}\;}-\bar{p}\tan\psi$$

where ϵ represents the eccentricity of the plastic potential surface; $\sigma_{to}\;$denotes the uniaxial tensile strength at failure; ψ is the dilation angle in the $\bar{p}$–$\bar{q}$ plane. 

The corresponding yield surface is calculated using Equation (11), expressed in terms of the effective stress that governs the failure or damage state.

(11) $$F=\frac{1}{1-\alpha }\left(\bar{q}-3\alpha \bar{p}+\beta \left({\tilde{\varepsilon }}^{pl}\right)\langle {\hat{\sigma }}_{max}\rangle -\gamma \langle -{\hat{\sigma }}_{max}\rangle \right)-{\bar{\sigma }}_{c}\left({\tilde{\varepsilon }}_{c}^{pl}\right)=0\;$$

where ${\hat{\sigma }}_{max}$ denotes the maximum principal effective stress; ${\tilde{\varepsilon }}_{t}^{pl}$ and ${\tilde{\varepsilon }}_{c}^{pl}$ are referred to as the equivalent plastic strains (${\tilde{\varepsilon }}^{pl}$) in tension and compression, respectively. The coefficients α, β and γ are computed using Equations (12)–(14)

(12) $$\alpha =\frac{\left(\sigma_{bo}/\sigma_{co}\right)-1}{2\left(\sigma_{bo}/\sigma_{co}\right)-1}$$

(13) $$\beta =\frac{{\bar{\sigma }}_{c}\left({\tilde{\varepsilon }}_{c}^{pl}\right)}{{\bar{\sigma }}_{t}\left({\tilde{\varepsilon }}_{t}^{pl}\right)}\left(1-\alpha \right)-\left(1+\alpha \right)$$

(14) $$\gamma =\frac{3\left(1-K_{c}\right)}{2K_{c}-1}$$

The ratio $\sigma_{bo}/\sigma_{co}$, representing the initial biaxial to uniaxial compressive yield stress, is typically assumed to be 1.16, resulting in a value of α=0.12. The parameter β is calculated as the ratio of the effective compressive cohesion stress $\left({\bar{\sigma }}_{c}\left({\tilde{\varepsilon }}_{c}^{pl}\right)\right)$ to the effective tensile cohesion stress $\left({\bar{\sigma }}_{t}\left({\tilde{\varepsilon }}_{t}^{pl}\right)\right)$. The parameter $K_{c}$ is defined as the ratio of the second deviatoric stress invariant on the tensile meridian (TM) to that on the compressive meridian (CM), as illustrated in Figure 6, and its default value is set to 2/3 [33,34].

The dilation angle is defined as the ratio of volumetric strain to shear strain and is typically set between 20° and 40° for concrete. This parameter significantly influences the overall behavior of the model as it governs the ductility of the material. An increase in the dilation angle leads to greater ductility in the system. Although the ABAQUS manual does not provide a specific recommended value for the dilation angle, previous studies [1] reported that a dilation angle of 50° yielded optimal results for preliminary analyses of PT structures. Therefore, a dilation angle of 50° was adopted for all numerical simulations in this study. The flow potential eccentricity is set to the default value of 0.1. A larger eccentricity increases the curvature of the potential surface, while a value that is very small may result in numerical convergence issues when confining stresses are insufficient. The viscosity parameter is set to zero by default in the ABAQUS/CAE 2024 indicating that viscoplastic regularization is not applied. However, assigning an appropriate value to this parameter can enhance convergence in the softening regime and improve the overall stability of the analysis. Finally, CDP model parameters adopted in this study based on the above considerations are presented in Table 4.

#### 3.3.2. Prestressing Tendon and Rebar Modeling

**Figure 7:**
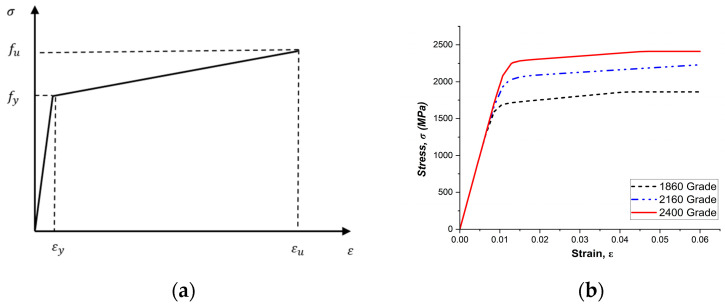
Stress–strain models: (**a**) rebar; (**b**) Prestressing Tendon.

In this study, the high-strength prestressing tendons were modeled using the Modified Ramberg–Osgood model, as illustrated in Figure 7b and defined by Equation (15) [48].

(15) fps=EεpfA+1−A1+Bεpfc1c ≤ fpu

Stress–strain behavior of high-strength prestressing strands cannot be accurately represented by simplified models such as the bi-linear or elastic–perfectly plastic formulations. Therefore, a mathematical model that can closely approximate the actual nonlinear behavior of such high-strength materials is required. Modified Ramberg–Osgood model is widely used for this purpose, as it can describe the transition between two linear regions through a smooth curve, making it particularly suitable for materials that do not exhibit a distinct yield point.

The model equation is given in Equation (15), where the coefficient A governs the slope of the post-yield region, B corresponds to the yield strain, and C controls the curvature of the transition zone between the elastic and inelastic responses. The constants (A, B, C) used for the prestressing tendons employed in this study are summarized in Table 5. In contrast, the rebar reinforcement was assumed to follow an idealized elastic–perfectly plastic behavior, as shown in Figure 7a. These stress–strain relationships were implemented in ABAQUS, and material nonlinearity was simulated using classical metal plasticity theory with the assumption of a von Mises yield surface [49].

## 4. Model Verifications and Numerical Results

### 4.1. Crack Pattern

**Figure 8:**
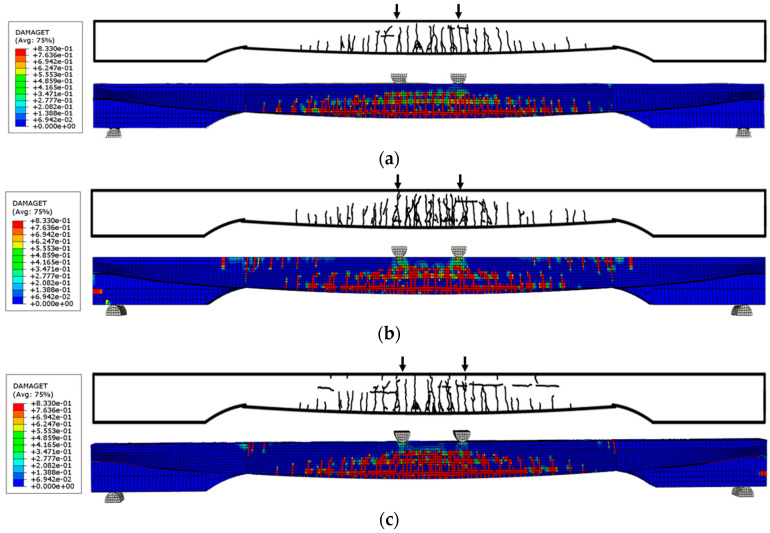
Comparison of numerical simulation damage patterns and experimentally observed crack patterns: (**a**) B-Type Girder; (**b**) C-Type Girder; (**c**) D-Type Girder.

The experimental crack map at failure with the FE-predicted crack distribution is compared in Figure 8. Under four-point bending, the first flexural cracks initiated at the midspan and multiplied between the two loading points; as loading magnitude increased, the dominant cracks developed predominantly in the vertical direction. With increasing load, some cracks propagated upward and coalesced beneath the loading points, and compressive crushing formed at the top concrete section within the constant-moment region at ultimate. The observed crack pattern was nearly symmetric about the span centerline, and no dominant web-shear behavior was identified. The FE results qualitatively reproduced the locations and orientations of the principal cracks and the extent of the compressive damage zone at the top surface, showing good agreement with the test observations.

### 4.2. Load-Deflection Response

**Figure 9:**
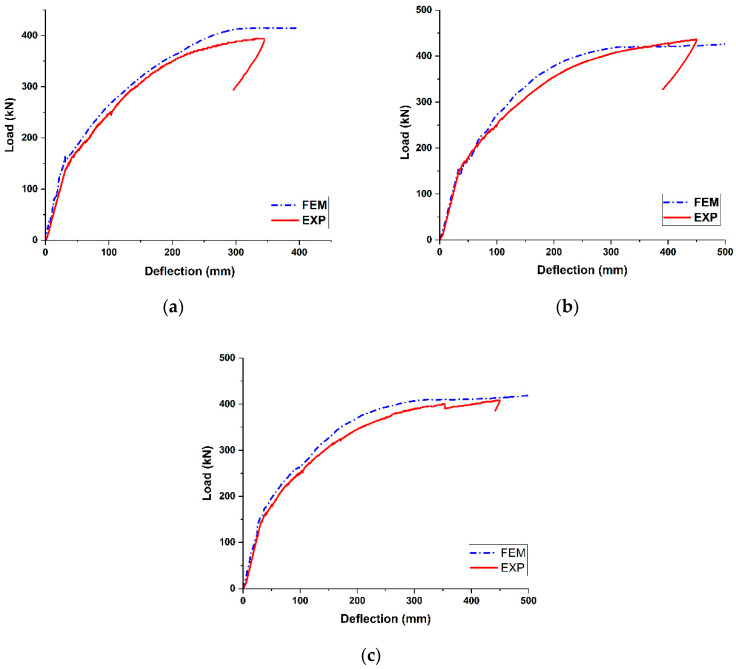
Load-Deflection comparison of numerical and test result: (**a**) SWPC 7B (B-type, 1860 MPa); (**b**) SWPC 7C (C-type, 2160 MPa); (**c**) SWPC 7D (D-type, 2400 MPa).

The experimental–numerical comparison on the global response of the girders is presented in Figure 9 in terms of force–displacement curves. Within the range of experimentally investigated displacements, the numerical results showed overall good agreement with the experimental findings. Table 6 summarizes the comparison details of the cracking load, its corresponding deflection, and the maximum load. It should be noted that, during the experimental observation of the ultimate flexural behavior of the PSC girders, the test was interrupted when the girders reached the ductile stage due to the safety concerns associated with tendon rupture. Therefore, in this study, the load corresponding to the final measured deflection in the experiment was defined as the maximum load ($P_{max}$) and used for comparison. The ratios of experimental to numerical results for the first cracking load were 0.91, 0.98, and 0.94 for the B-, C-, and D-type girders, respectively. The corresponding ratios of deflection at the first cracking load were 1.09, 1.03, and 0.99, while the ratios for the maximum load were 0.95, 1.03, and 0.97, respectively. These differences are within the acceptable tolerance range and can be attributed to variations between the experimental environment and the modeling assumptions.

Table 7 presents the comparison details of the initial and post-cracking stiffness of the girders. The ratios of experimental to numerical results for the initial stiffness ranged from 0.83 to 0.94, whereas those for the post-cracking stiffness ranged from 0.97 to 1.08. The stiffness values obtained from the FE model were slightly higher than those from the experiments, which can be explained by the modeling assumption of a fully bonded interaction between the prestressing tendons and concrete. This assumption led to an overestimation of the tension stiffening effect, since the FE model enforced complete strain compatibility between the concrete and reinforcement even after cracking. Nevertheless, the differences in cracking and maximum loads between FEM and experimental results were within the general tolerance limits, which can be reasonably explained by deviations between the modeling assumptions and real structural behavior.

## 5. Parametric Study

In the proposed parametric study, the flexural behavior of post-tensioned prestressed concrete (PSC) girders was investigated by varying key factors that influence long-term resistance and serviceability. The considered parameters included tendon tensile strength (1860, 2160, and 2400 MPa), residual effective prestress corresponding to different service lives (0, 25, 50, and 100 years), concrete compressive strength, tendon cross-sectional area, and the presence of grouting defects. All other geometric details, analysis procedures, interaction models, and boundary conditions were kept consistent with the validated model presented in Section 3.

A total of sixteen girders were included in the parametric study, which were divided into two groups. Group 1 comprised twelve girders designed to evaluate the combined influence of tendon tensile strength and service life. Three tendon strength grades (1860, 2160, and 2400 MPa) were considered, and for each grade, four service lives (0, 25, 50, and 100 years) were modeled to account for long-term prestress losses. Group 2 consisted of four girders with identical tendon strength (1860 MPa) and service life (100 years), but with different parameters varied individually: (i) reduced concrete compressive strength, (ii) presence of grouting defects, (iii) reduced tendon cross-sectional area, and (iv) combined defects including all three conditions simultaneously.

The specific variables adopted in the parametric study are summarized in Table 8, which presents the effective prestress ($P_{e}$) corresponding to each service life, the design concrete compressive strength ($f_{cu}$), the presence or absence of grouting defects, and the tendon cross-sectional area. The focus of Group 1 was to quantify the reduction of residual prestress over time and its effect on flexural strength and stiffness, while Group 2 aimed to assess the sensitivity of girders to construction defects and material deterioration under long-term conditions.

## 6. Results and Discussion

The numerical analyses conducted in this study focused on investigating the flexural behavior of post-tensioned PSC girders prestressed with high-strength tendons under positive bending moments. The parametric study examined the influence of several key variables, including the residual effective prestress associated with different service lives, the design compressive strength of concrete, the presence of grouting defects, and the tendon cross-sectional area. To reasonably represent both serviceability and ultimate limit states, the flexural load capacities corresponding to girder deflections of L/100 and L/50 were derived, herein denoted as $P_{L/100}$ (flexural load capacity at L/100 deflection) and $P_{L/50}$ (flexural load capacity at L/50 deflection), respectively. The results obtained from this parametric study were subsequently compared and analyzed across the different variables and cases, thereby evaluating the long-term behavior of PSC girders as well as the influence of potential defects arising from aging and deterioration on their structural response.

### 6.1. Influence of Residual Prestressing Force (Service Life)

**Figure 10:**
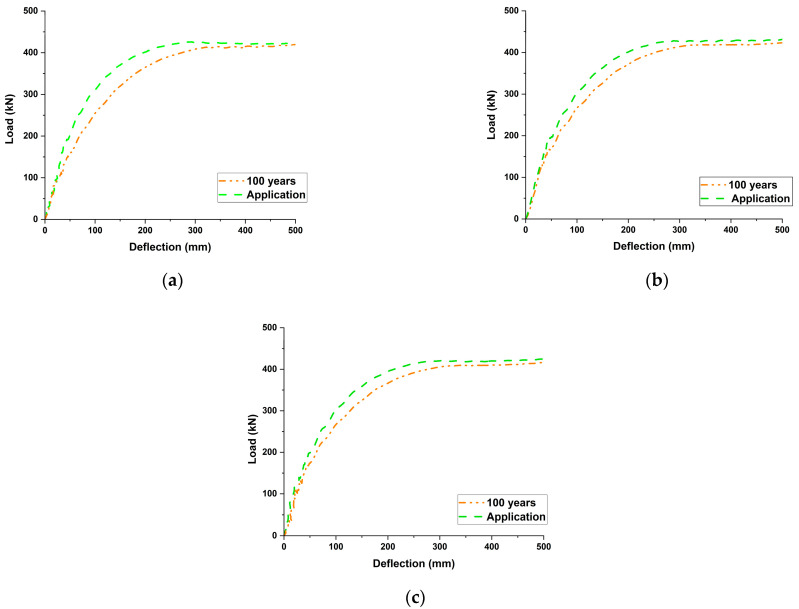
Load-Deflection comparison at prestress application and at 100 years: (**a**) SWPC 7B (B-type, 1860 MPa); (**b**) SWPC 7C (C-type, 2160 MPa); (**c**) SWPC 7D (D-type, 2400 MPa).

**Figure 11:**
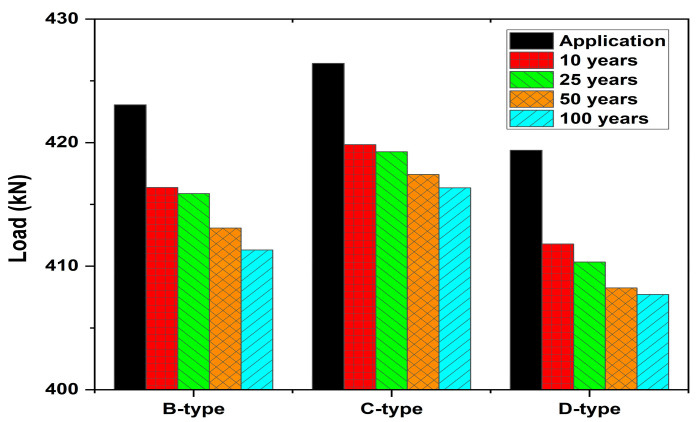
Comparison of Ultimate Load Capacities of PSC Girders with Different Service Life.

In this parametric study, the influence of residual prestressing force ($P_{e}$) on the flexural behavior of PSC girders was systematically analyzed. Residual effective prestress values corresponding to service lives of 0, 25, 50, and 100 years were applied. Among these, the 0-year value was taken from the actual introduced prestressing force at the time of construction, whereas the values for 25, 50, and 100 years were derived using the long-term loss prediction equation proposed by Park et al. [15] (Equations (1) and (2)). These values were then used to evaluate changes in stiffness, deflection, and ultimate load. Table 9 summarizes the numerical results, Figure 10 illustrates the load–deflection curves according to the residual prestress levels, and Figure 11 presents the variation in ultimate load with increasing service life for each tendon type.

Residual prestressing force exhibited a clear reduction with prolonged service life. For example, in the case of B-type tendons, $P_{e\;}$ decreased from 1333.2 MPa at the initial stage to 962.8 MPa after 100 years, representing a reduction of approximately 28%. This prestress loss had a direct impact on the early portion of the load–deflection response, resulting in reduced stiffness and increased deflection. Specifically, for B-type girders, the load at L/100 deflection decreased from 391.9 kN to 349.9 kN, corresponding to a reduction of about 10.8%. Similarly, C- and D-type girders showed reductions of 8.1% and 7.9%, respectively, indicating that prestress loss over time significantly affects the serviceability limit state performance of PSC girders.

In contrast, the effect on ultimate load was relatively limited. As shown in Table 9 and Figure 10, for Group 1, the reduction rate of ultimate load at L/50 deflection was less than 3%. B-type girders decreased from 423.1 kN to 411.3 kN (−2.8%), C-type from 426.4 kN to 416.3 kN (−2.4%), and D-type from 419.4 kN to 408.2 kN (−2.6%). Therefore, while long-term prestress losses play a major role in stiffness and deflection behavior at the serviceability limit state, their influence on the ultimate flexural strength is highly limited.

These results can be interpreted structurally as follows: In the initial and post-cracking stages, prestressing introduces additional compressive force into the section, delaying crack initiation and suppressing deflection. Accordingly, when prestress loss occurs over time, cracking initiates earlier and both initial stiffness and deflection behavior deteriorate significantly. However, once the section undergoes plasticization and reaches the ultimate state, the tendon stress approaches limiting values such as $f_{p,\;1\%}$ or $f_{pu}$, regardless of the initial prestress level. Consequently, long-term prestress loss is a critical factor governing serviceability performance, but its impact on the final ultimate flexural resistance remains minimal.

### 6.2. Influence of Grouting Defect

**Figure 12:**
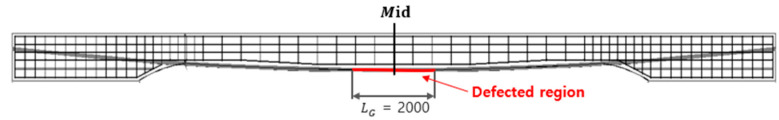
Grouting condition of girder (defected region in red): G14-B-100Y-GD.

In PSC girders, grouting defects may develop over time due to incomplete filling, bleeding, or microcracking. In this study, a simplified modeling approach was employed to evaluate the influence of such defects on the flexural behavior. Figure 12 illustrates the analysis condition where a grouting defect was introduced over a 2000 mm region at midspan, representing the case in which a defect occurs in the zone most sensitive to flexural response.

**Figure 13:**
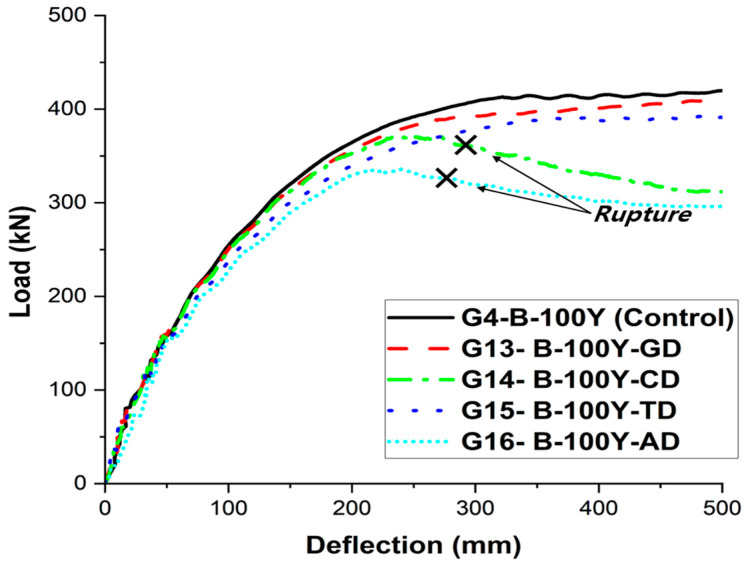
Load–Deflection Response of PSC Girders under Different Material and Defect Conditions (Group 2).

As shown in the load–deflection curves in Figure 13, the girder with a grouting defect (G14-B-100Y-GD) exhibited lower load resistance throughout the entire response compared with the reference girder without defects (G4-B-100Y, Control). According to Table 9, the ultimate load decreased from 411.3 kN to 397.5 kN, corresponding to a reduction of approximately 3.4%. This behavioral degradation can be attributed to the loss of confinement effect in the defect region, where the tendon was no longer embedded in concrete, resulting in the disappearance of the tension stiffening effect in that zone.

### 6.3. Influence of Concrete Strength Degradation

The influence of concrete strength degradation on the flexural response of PSC girders was quantified. In actual structures, long-term deterioration mechanisms such as carbonation, chloride ingress, and micro-cracking can reduce the concrete compressive strength. Prior studies have reported that, under severe environmental deterioration and prolonged exposure, the concrete compressive strength typically decreases by approximately 20% [50,51,52,53]. Accordingly, this study adopted a deterioration scenario by reducing the design compressive strength ($f_{cu}$) of the reference girder (G4-B-100Y) from 40 MPa to 32 MPa (−20%), while keeping all other analysis parameters identical to those of the reference model.

The results presented in Table 9 and Figure 13 clearly demonstrate the adverse effects of reduced concrete strength on girder behavior. At the serviceability stage (L/100), the differences remained relatively small, whereas at the ultimate state, unlike the reference girder, the deteriorated girder (G13-B-100Y-CD) experienced premature compressive failure (rupture) before reaching the ductile range. The load–deflection curve in Figure 13 also shows that the girder failed before entering the post-peak region, with a sudden drop in load capacity, indicating significant losses in both strength and ductility. This behavior can be attributed to the reduction of concrete compressive capacity, which decreases the effective depth of the compression block and residual ductility, causing the concrete to reach its ultimate strain more rapidly under the same external loading and prestressing conditions.

### 6.4. Influence of Tendon Cross-Section Reduction

Long-term corrosion in PSC girders can reduce the cross-sectional area of tendons, directly impairing their flexural resistance. Previous studies have reported that the extent of tendon section loss due to corrosion generally ranges from 0% to 20%, and Seoul Metropolitan Facilities Management Corporation (SMFMC) highlighted that tensile strength and elongation decrease significantly when the loss exceeds 10–20% [54,55,56,57]. Reflecting these findings, this study adopted a simplified approach by assuming a 10% reduction in tendon cross-sectional area to evaluate its influence on the flexural performance of PSC girders.

**Figure 14:**
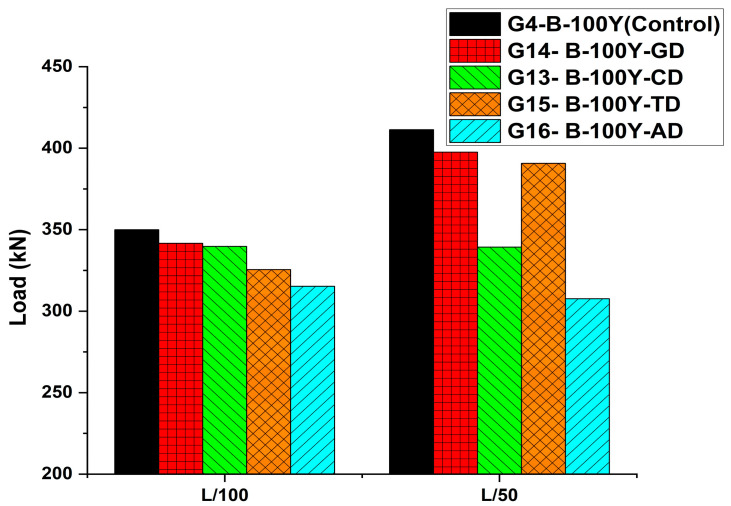
Ultimate Load Comparison of PSC Girders with Material and Defect Variations (Group 2).

According to the results presented in Table 9 and Figure 13 and Figure 14, the reduction of tendon cross-section significantly weakened the load-carrying capacity and overall behavior of the PSC girders. At the serviceability stage (L/100), the load of the reference girder (G4-B-100Y) was 349.9 kN, whereas the tendon-reduced girder (G15-B-100Y-TD) carried only 325.5 kN, corresponding to a decrease of about 7.0%. At the ultimate state (L/50), the load decreased from 411.3 kN to 390.5 kN, indicating a reduction of approximately 5.1%. The load–deflection response shown in Figure 13 further supports this trend. The tendon-reduced girder exhibited lower stiffness than the reference girder throughout the entire loading range, and its load resistance was diminished even at the ultimate stage. This behavior can be attributed to the reduction in prestressing force induced by the decreased tendon cross-sectional area, which consequently weakened the composite action between the tendon and the surrounding concrete.

### 6.5. Influence of Combined Defects

The girder G16-B-100Y-AD was modeled by simultaneously incorporating concrete strength degradation, tendon cross-sectional reduction, and grouting defects. As shown in Figure 13 and Figure 14, this girder exhibited the most vulnerable behavior among all cases. Premature failure (rupture) occurred before reaching the ductile range, preventing the girder from achieving a typical ultimate state. The load–deflection curve also revealed a sharp drop in load capacity without entering the post-peak region, indicating severe losses in both strength and ductility.

## 7. Conclusions

This parametric study was conducted using finite element analysis (FEA) to evaluate the long-term flexural behavior of prestressed concrete (PSC) girders under various degradation scenarios, including prestress loss, grouting defects, concrete strength reduction, and tendon cross-sectional loss. The main conclusions are as follows.

1. The long-term performance of PSC girders is strongly affected by prestress loss at the serviceability limit state, whereas ultimate strength is more sensitive to material and cross-sectional deterioration. The coexistence of multiple degradation factors accelerates performance loss, underscoring the need to incorporate such effects into life-cycle performance evaluation and maintenance planning.
2. The numerical model was validated against experimental results, showing reasonable agreement in terms of crack initiation, stiffness transition, and maximum load, with errors remaining within acceptable limits. This confirms that the developed model can be reliably applied to long-term degradation scenarios.
3. Residual prestress (service life) had a significant influence on the serviceability behavior of PSC girders. As service life increased, effective prestress decreased, leading to reduced stiffness, increased deflections, and earlier crack initiation. However, the influence on ultimate flexural capacity was relatively minor, indicating that long-term performance degradation is primarily pronounced at the serviceability limit state.
4. Girders with grouting defects exhibited reduced load resistance throughout the entire load–deflection response. This reduction is attributed to the loss of bond and confinement effects in the defect zone, where the tendons were no longer embedded in the concrete, thereby eliminating the tension stiffening effect.
5. In the case of reduced concrete compressive strength, premature failure occurred before sufficient ductility could be developed. This behavior was due to the rapid reduction in compressive capacity and residual ductility, which caused the concrete to reach its ultimate strain more quickly under the same external loading and prestress conditions. Consequently, both strength and ductility losses were pronounced.
6. Girders with reduced tendon cross-sectional area exhibited lower stiffness and strength across the entire load–deflection response compared to the reference girder. This was primarily caused by reduced prestressing force and a weakened composite action between tendon and concrete, thereby diminishing both serviceability and load-bearing capacity.
7. The girder subjected to combined degradation exhibited the most critical behavior, failing prematurely before ductility could develop. The strength loss ratio was the largest among all cases, confirming that the accumulation of multiple deterioration factors accelerates structural performance degradation. This highlights the necessity of accounting for compound deterioration effects in the durability assessment and maintenance strategies of PSC girders.
8. This study is limited by simplified modeling that treats concrete deterioration solely as a reduction in strength and neglects tendon slip and time-dependent corrosion, and future work should adopt advanced coupled models and long-term field validation to capture deterioration more faithfully and support life cycle assessment and performance-based maintenance strategies for PSC girders.

## Figures and Tables

**Table 1:** Comparison of calculated and measured values.

Time	Modified Calculated Prestress Value			Measured Prestress Value		
	B-Type (MPa)	C-type (MPa)	D-Type (MPa)	B-Type (MPa)	C-Type (MPa)	D-Type (MPa)
2015.08	1106.90	1349.72	1542.11	1128.85	1379.33	1538.23
2016.08	1058.26	1306.56	1501.94	1079.26	1289.33	1502.58
2017.08	1038.68	1288.97	1486.37	1067.87	1282.16	1487.34
2018.08	1027.77	1279.03	1476.50	1060.20	1293.00	1476.36
2019.08	1021.22	1272.53	1469.57	1032.00	1267.47	1461.78
2020.08	1015.76	1268.22	1465.36	1031.68	1294.45	1464.64
2021.08	1012.01	1264.34	1461.52	1023.21	1282.10	1459.65
2022.08	1009.06	1261.52	1458.92	1012.33	1280.79	1474.12
2023.08	1006.64	1259.18	1456.35	1011.66	1275.83	1465.42

**Table 2:** Experimental load-deflection results and stiffness of girders.

ID	$P_{cr}$ (kN)	${∆}_{cr}$ (mm)	$P_{max}$ (kN)	${∆}_{max}$ (mm)	$K_{i}$ Initial	$K_{p}$ Post Cracking
B-type	139.50	31.86	393.9	344.46	4.38	0.81
C-type	150.30	33.75	436.80	449.45	4.46	0.69
D-type	151.05	34.71	408.05	449.94	4.35	0.62

**Table 3:** Tendon Specifications for three Tensile Strengths.

Type of Tendon	SWPC 7B (B-Type)	SWPC 7C (C-Type)	SWPC 7D (D-Type)
Tensile Strength (MPa)	1860	2160	2400
Diameter (mm)	15.2	15.2	15.2
$\mathrm{Cross}-\mathrm{Sec}\mathrm{tional}\;\mathrm{area}\;({mm}^{2}$)	138.7	138.7	138.7
Unit Weight (kg/m)	1.101	1.101	1.101
Yield Load (kN)	222	255	283
Ultimate Load (kN)	261	300	333
Yield Strength (MPa)	1600	1830	2040
Ultimate Strength (MPa)	1860	2160	2400
Elastic Modulus (MPa)	200,000	200,000	200,000
Initial Prestressing Stress (MPa)	1333	1550	1730
Residual Prestressing Stress after 10 Years of Loss (MPa)	1004	1256	1458
$V=f_{pi,L}/f_{pi,H}$	1	0.86	0.77

**Table 4:** Plasticity parameters of the concrete model.

Density (kg/m^3^)	Elastic Modulus (MPa)	Poisson Ratio	Dilation Angle	Eccentricity	fbo/fco	KC	Viscosity Parameter
2350	33,346	0.167	50	0.1	1.16	0.6667	0

**Table 5:** The constants of Modified Ramberg-Osgood model for high-strength strands.

Properties	Tensile Strength of Strand (MPa)		
	1860	2160	2400
Modulus of elasticity (MPa)	200,000	200,000	200,000
Constant A	0.025	0.017	0.020
Constant B	118	97	88
Constant C	10	8	13

**Table 6:** Experimental and numerical results of cracking load, cracking deflection, and max load.

Girder ID	Experimental			Numerical			Experimental/Numerical		
	$P_{cr}^{e}$ (kN)	${∆}_{cr}^{e}$ (mm)	$P_{max}^{e}$ (kN)	$P_{cr}^{n}$ (kN)	${∆}_{cr}^{n}$ (mm)	$P_{max}^{n}$ (kN)	$P_{cr}^{e}/P_{cr}^{n}$	${∆}_{cr}^{e}$ $/{∆}_{cr}^{n}$	$P_{max}^{e}/P_{max}^{n}$
B-type	139.50	31.86	393.90	153.40	29.23	416.09	0.91	1.09	0.95
C-type	150.30	33.75	436.80	153.78	32.70	422.79	0.98	1.03	1.03
D-type	151.05	34.71	408.05	156.11	34.88	418.57	0.94	0.99	0.97

**Table 7:** Experimental and numerical results of initial flexural stiffness, post cracking flexural stiffness.

Girder ID	Experimental		Numerical		Experimental/Numerical	
	$K_{i}$ Initial	$K_{p}$ Post Cracking	$K_{i}$ Initial	$K_{p}$ Post Cracking	$K_{i}$ Initial	$K_{p}$ Post Cracking
B-type	4.38	0.81	5.25	0.83	0.83	0.97
C-type	4.46	0.69	4.70	0.64	0.95	1.08
D-type	4.35	0.62	4.62	0.63	0.94	0.98

**Table 8:** Parametric study variables for Numerical simulation of PSC girders (Grouting defect: N = no, Y = yes).

Group	ID	Tendon-Type (MPa)	Service Life (Year)	$P_{e}$(MPa)	$f_{cu}$(MPa)	Grouting Defect	Tendon Cross-Section $\left({\mathrm{mm}}^{2}\right)$
Group 1	G1-B-0Y	1860	0	1333.23	40	N	138.7
	G2-B-25Y	1860	25	989.35	40	N	138.7
	G3-B-50Y	1860	50	977.37	40	N	138.7
	G4-B-100Y	1860	100	962.83	40	N	138.7
	G5-C-0Y	2160	0	1550	40	N	138.7
	G6-C-25Y	2160	25	1241.61	40	N	138.7
	G7-C-50Y	2160	50	1229.34	40	N	138.7
	G8-C-100Y	2160	100	1214.57	40	N	138.7
	G9-D-0Y	2400	0	1730	40	N	138.7
	G10-D-25Y	2400	25	1443.10	40	N	138.7
	G11-D-50Y	2400	50	1430.6	40	N	138.7
	G12-D-100Y	2400	100	1415.65	40	N	138.7
Group 2	G13-B-100Y-CD	1860	100	962.83	32	N	138.7
	G14-B-100Y-GD	1860	100	962.83	40	Y	138.7
	G15-B-100Y-TD	1860	100	962.83	40	N	124.83
	G16-B-100Y-AD	1860	100	962.83	32	Y	124.83

**Table 9:** Parametric study results for Numerical simulation of PSC girders.

Group	ID	$P_{L/100}$ (kN)	$P_{L/50}$ (kN)	$\frac{P_{L/100}}{P_{L/100}^{0\;year}}$	$\frac{P_{L/50}}{P_{L/50}^{0\;year}}$	Strength Loss Ratio (%)
Group 1	G1-B-0Y	391.91	423.06	1.00	1.00	0
	G2-B-25Y	365.16	415.87	0.931	0.983	1.7
	G3-B-50Y	353.14	413.08	0.901	0.976	2.4
	G4-B-100Y	349.93	411.31	0.892	0.972	2.8
	G5-C-0Y	389.36	426.41	1.00	1.00	0
	G6-C-25Y	361.38	419.26	0.928	0.983	1.7
	G7-C-50Y	360.34	417.42	0.925	0.978	2.2
	G8-C-100Y	358.10	416.34	0.919	0.976	2.4
	G9-D-0Y	383.15	419.38	1.00	1.00	0
	G10-D-25Y	355.65	410.33	0.928	0.978	2.2
	G11-D-50Y	354.42	408.24	0.925	0.973	2.7
	G12-D-100Y	353.25	407.70	0.921	0.972	2.8
Group 2	G13-B-100Y-CD	339.69	339.23 (Rupture)	0.970	0.824	17.6
	G14-B-100Y-GD	341.62	397.53	0.976	0.966	3.4
	G15-B-100Y-TD	325.52	390.51	0.930	0.949	5.1
	G16-B-100Y-AD	315.24	307.60 (Rupture)	0.900	0.747	25.3

## Data Availability

The original contributions presented in this study are included in the article. Further inquiries can be directed to the corresponding author.
